# Serendipitous discovery of light-induced (*In Situ*) formation of an Azo-bridged dimeric sulfonated naphthol as a potent PTP1B inhibitor

**DOI:** 10.1186/s12858-017-0083-3

**Published:** 2017-05-31

**Authors:** Robert D. Bongard, Michael Lepley, Khushabu Thakur, Marat R. Talipov, Jaladhi Nayak, Rachel A. Jones Lipinski, Chris Bohl, Noreena Sweeney, Ramani Ramchandran, Rajendra Rathore, Daniel S. Sem

**Affiliations:** 1 Center for Structure-based Drug Design and Development, Department of Pharmaceutical Sciences, Concordia University of Wisconsin, Mequon, WI 53097 USA; 2 Department of Pediatrics, Division of Neonatology, Department of Obstetrics and Gynecology, Children’s Research Institute (CRI) Developmental Vascular Biology Program, Translational and Biomedical Research Center, 8701 Watertown Plank Road, P.O. Box 26509, Milwaukee, WI 53226 USA; 3 Department of Chemistry, Marquette University, Wehr Chemistry Building, P.O. Box 1881, 535 N. 14th Street, Milwaukee, WI 53201 USA

**Keywords:** DUSP5, *p*ERK, PTP1B, Azo, Dyes, Enzyme kinetics

## Abstract

**Background:**

Protein tyrosine phosphatases (PTPs) like dual specificity phosphatase 5 (DUSP5) and protein tyrosine phosphatase 1B (PTP1B) are drug targets for diseases that include cancer, diabetes, and vascular disorders such as hemangiomas. The PTPs are also known to be notoriously difficult targets for designing inihibitors that become viable drug leads. Therefore, the pipeline for approved drugs in this class is minimal. Furthermore, drug screening for targets like PTPs often produce false positive and false negative results.

**Results:**

Studies presented herein provide important insights into: (a) how to detect such artifacts, (b) the importance of compound re-synthesis and verification, and (c) how *in situ* chemical reactivity of compounds, when diagnosed and characterized, can actually lead to serendipitous discovery of valuable new lead molecules. Initial docking of compounds from the National Cancer Institute (NCI), followed by experimental testing in enzyme inhibition assays, identified an inhibitor of DUSP5. Subsequent control experiments revealed that this compound demonstrated time-dependent inhibition, and also a time-dependent change in color of the inhibitor that correlated with potency of inhibition. In addition, the compound activity varied depending on vendor source. We hypothesized, and then confirmed by synthesis of the compound, that the actual inhibitor of DUSP5 was a dimeric form of the original inhibitor compound, formed upon exposure to light and oxygen. This compound has an IC_50_ of 36 μM for DUSP5, and is a competitive inhibitor. Testing against PTP1B, for selectivity, demonstrated the dimeric compound was actually a more potent inhibitor of PTP1B, with an IC_50_ of 2.1 μM. The compound, an azo-bridged dimer of sulfonated naphthol rings, resembles previously reported PTP inhibitors, but with 18-fold selectivity for PTP1B versus DUSP5.

**Conclusion:**

We report the identification of a potent PTP1B inhibitor that was initially identified in a screen for DUSP5, implying common mechanism of inhibitory action for these scaffolds.

**Electronic supplementary material:**

The online version of this article (doi:10.1186/s12858-017-0083-3) contains supplementary material, which is available to authorized users.

## Background

Protein tyrosine phosphatases (PTPs) play a central role in cell biology, acting as a complement to the protein kinase system, to control levels of phosphorylated proteins in the cell. By the removal of the phosphate groups that are added by protein kinases, the PTPs regulate processes that range from cell growth and differentiation to motility to adhesion, in both normal and disease states [1, 2]. There are a wide variety of PTPs, and within the human genome 107 PTPs are classified into four classes. In this study, we focus on two class I PTPs. One is the cysteine-based PTPs, which are specific for phosphotyrosines. These are the so-called “classical PTPs,” and include PTP1B [3]. A second category of class I PTP is the dual specificity phosphatases (DUSPs). Our studies initially focused on targeting DUSP5, which in addition to phospho-tyrosine also dephosphorylates and phospho-threonine residue. Ultimately, through serendipity, we identified a DUSP5 inhibitor that was actually more potent and selective at inhibiting PTP1B as well as protein tyrosine phosphatase, non-receptor type 11 (SHP-2). The DUSPs, and their mitogen-activated protein kinase (MAPK) partners, are involved in various diseases, including cancer, diabetes, and autoimmune disorders [4–7]. DUSP5, in particular, has been shown by the Ramchandran lab as important for early vascular patterning in vertebrates, and a clinically relevant serine to proline mutation (S147P) has been identified in patients with vascular anomalies [8]. Recently, it was shown using DUSP5 knockout mice that DUSP5 negatively regulates interleukin-33-mediated eosinophil survival and function, [9], and upon viral infection, DUSP5 is essential for T cell survival [10]. Furthermore, DUSP5 knockout rats displayed autoregulation of cerebral blood flow [11]. Thus, DUSP5 is an important new drug target, and was pursued by the studies presented herein – at least until it was discovered that our lead compound was a more potent inhibitor of PTP1B.

PTP1B is a drug target for treatment of cancer [12, 13] as well as type II diabetes and obesity [14]. While there are presently no approved drugs targeting PTP1B [15], it has been hotly pursued as a drug target by many pharmaceutical companies and academic labs [15, 16] in the hope that an inhibitor of PTP1B could be developed as a drug for treating type II diabetes, which is anticipated to affect over 300 million people world-wide by 2030 [14, 17]. PTP1B works by dephosphorylating the insulin receptor, and studies with knockout mice have shown that decreased PTP1B activity is associated with weight loss and enhanced insulin sensitivity [18]. PTP1B was the first PTP to be cloned and characterized [19], and was first characterized by crystallography in 1994 [20]. Despite this extensive knowledge base, and the recognition of PTP1B’s important role as a drug target, there are still no approved drugs that target PTP1B. It has been suggested that there are unique challenges to developing drugs for PTPs, in part due to the requirement that inhibitors be highly charged, which creates bioavailability problems for drug lead molecules [16]. Adding to this challenge, it has been noted that a large number of small molecules identified as PTP inhibitors were later found to be false positives, that inhibit nonspecifically (e.g. hydrophobic; aggregation effects) or via oxidation of the active site cysteine residue [15]. Such screening artifacts are not unique to PTPs, and are a growing concern as increasing numbers of labs participate in drug discovery and development efforts.

The studies presented herein provide a useful case study concerning the purity and chemical identities of small molecules and their degradation, role of careful analysis of apparent screening artifacts such as time-dependent inhibition, and the importance of serendipity in drug discovery.

## Methods

### Preparation of RR535 and RR601

#### Commercial RR535

Synthesis of RR535 (in-house NCI2602 re-synthesis) was achieved in a multi-step synthesis only in very poor yield and therefore the protocol was abandoned. Fortunately, an acid derivative of RR535 (5-amino-1-naphthol-3-sulfonic acid hydrate, TCI) was commercially available. It was converted to RR535 by a reaction with equimolar sodium hydroxide in water under an inert atmosphere followed by precipitation by a layering technique with pure acetone. The resulting precipitate was further purified by repeated re-precipitation (3 times) with a mixture of water and layering with acetone to afford a pure sample of RR535.

#### Synthesis of RR601

**Scheme 1:**
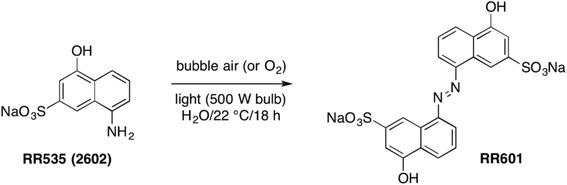
Synthesis of RR601

A solution of 5-amino-1-naphthol-3-sulfonic acid hydrate, TCI (1.0 g, 4.17 mmol) was added an aqueous solution of NaOH (0.17 g, 4.17 mmol) under an argon atmosphere. The resulting mixture was stirred vigorously and the pH of the solution was checked to ensure the complete conversion of the acid into RR535. Water was evaporated by bubbling a constant stream of argon through the solution for 12 h to yield RR535 in quantitative amount. The resulting RR535 (1 g) was re-dissolved in deionized water (10 mL) in a quartz tube equipped with a magnetic stirring bar. The resulting solution was vigorously stirred while a stream of air (or pure oxygen) was passed through the reaction mixture and the tube was exposed to a 120 W lamp for 18 h. The dark colored reaction mixture was slowly evaporated by bubbling argon through the solution at 45 **°**C to produce a black brown solid. This solid was further purified by repeated re-precipitation (5 times) from a mixture of water and acetone to afford a pure RR601 in 45% yield. NMR and mass spectrometry (MS) was used to characterize RR601 Scheme 1.

### In vitro ERK dephosphorylation western blot assay

GST-DUSP5 purified protein was generated using previously published methods [21]. The protein was diluted in phospho-ERK buffer (30 mM Tris-HCl pH 7.0, 75 mM NaCl, 0.67 mM EDTA, 1 mM DTT, H_2_O) to a concentration of 1.5–3.0 nM, depending on the purity. Active ERK2 (R&D Systems, Minneapolis MN) and the compounds to be tested were also diluted in this buffer with an initial concentration of 30 nM for ERK2 and serial dilutions for the compounds. 5 μL each of GST-DUSP5 and diluted compound concentrations were incubated for 5 mins after which 5 μL of 30 nM ERK2 was added and allowed to incubate for 20 mins. After this time 15 μL SDS-Loading buffer was added to each reaction. Samples were boiled for 5 mins, loaded into lanes of 12% Mini-Protean TGX gels (Bio-Rad Laboratories Inc, Hercules CA), and run at 120 V. Protein samples were then transferred to PVDF western blotting Membranes (Roche Diagnostics, Indianapolis IN) at 90 V for 1 h. Membranes were treated and utilized in the iBind Flex Western Device (Thermo Fisher Scientific, Waltham MA) according to manufacturer protocols. Membranes were probed for total and phospho-ERK using rabbit anti-human p44/42 MAPK and mouse anti-human phospho-p44/42 MAPK primary antibodies and HRP-linked anti-rabbit and anti-mouse secondary antibodies (Cell Signaling Technology Inc, Danvers MA). Images were developed using a FluorChem HD2 imager (Bio-Techne, Minneapolis MN) after application of SuperSignal West Femto and West Pico chemiluminescent substrate (Thermo Fisher Scientific).

### IC_50_ calculation from the western blot assay

Densitometry analysis of western blot images was performed using ImageJ software. IC_50_ values were obtained using Graphpad Prism 6 software to perform a non-linear least squares regression, which generated a sigmoidal dose-response curve. From this analysis IC_50_ values were obtained for each inhibitor being tested: NCI2602 (Additional file 1: Fig. S2), RR535 (Additional file 1: Fig. S3) and RR601 (Additional file 1: Fig. S4). In contrast to the IC_50_ assay determined in solution and described in the next section, this assay was done with full-length protein (containing both domains), and using *p*ERK as substrate. Thus, it is expected that slightly different IC_50_ values might be obtained, since the conformation of the active site is expected to be affected by the presence of the ERK binding domain, in the presence of *p*ERK.

### DUSP5 phosphatase domain protein synthesis and details of *p*NPP assay

The DUSP5 phosphatase domain (DUSP5 PD(WT)) gene was synthesized by Blue Heron (Bothell, WA) and the protein expressed and purified as previously described [22]. To measure the enzymatic activity of wild type phosphatase domain of DUSP5 {DUSP5 PD(WT)} and the inhibitory capacity of selected compounds, an in vitro phosphatase assay was utilized as previously described [22]. Briefly, assays without and with inhibitors were performed in Greiner 96-well clear bottom plates with a total assay volume of 200 μL. The assay buffer contained 100 mM Tris, 100 mM NaCl, 5 mM MgCl_2_ • 6H_2_O and 1 mM dithiothreitol (DTT) at pH 7.5. *p*-nitrophenyl phosphate (*p*NPP, Sigma Aldrich) 5 mM was used as the substrate. DUSP5 PD(WT) dephosphorylates *p*NPP to yield *p*-nitrophenolate, which absorbs at 405 nm, having an extinction coefficient of 18,000 M^-1^ cm^-1^. The reaction was initiated by the addition of 4 μL of a 29 μM DUPSP5 PD(WT) enzyme stock and absorbance was monitored at 25 °C over time using a Spectramax M5 microplate reader (Molecular Devices). Blanks contained only buffer and *p*NPP. Negative controls (without inhibitor) contained assay buffer with *p*NPP and DUSP5 PD(WT).

### Inhibition of DUSP5PD(WT) by NCI2602 (1-amio-5-napththol-7-sulfonic acid), and effects of compound source and storage conditions

The first small molecule inhibitor tested was procured from three independent sources. First from the NCI Diversity Chemical Library (NCI2602), then from a commercial vendor (MP Biomedicals, Cat. No. 05211488, CAS 489-78-1), and was synthesized in our laboratories (RR535). These compounds should, in theory, all be the same chemical structure, 1-amio-5-napththol-7-sulfonic acid. Stock solutions (25 mM) of each inhibitor compound, including RR601 (newly synthesized) and NSC-87877 (Merck Millipore) were prepared in dimethyl sulfoxide (Sigma-Aldrich). We first noticed a time-dependent darkening of the stock solutions of NCI2602 stored in room light as well as stock solutions of RR535 and the MP Biomedicals compound that had been exposed to room light for varying amounts of time. This initial observation prompted us to study the effect of light exposure and compound source on inhibitor potency. Upon the addition of 4 μL of these respective inhibitor stock solutions to the assay buffer, the resulting range in assay inhibitor concentrations were 0.1 μM to 1,000 μM for the various 1-amino-5-naphthol-7-sulfonic acid sources, 0.1 μM to 300 μM for RR601 and 0.1 μM to 300 μM for NSC-87877. Appropriate vehicles were added to the blank and negative control wells. A minimum of three replicate wells was run for each condition and at each inhibitor concentration. The replicate wells were averaged to give a single data point. The reaction was initiated upon addition of 4 μL of a 29 μM enzyme stock which was dispensed to each of the wells except the blanks utilizing a single channel pipette. The resulting DUSP5 PD(WT) concentration in the assay buffer was 0.58 μM. The plate was returned to the plate reader, shaken, and absorbance recorded at 25 °C every thirty seconds over a ten to sixty mins time course. In selected experiments the plate was returned to the plate reader following a 10 mins recording for one or two additional 10 mins absorbance recordings.

### RR601 and NSC-87877 inhibition of PTP1B and SHP-2 activity

To examine the capacity of RR601 to inhibit protein-tyrosine phosphatase 1B (PTP1B), (Creative Biomart), activity, an in vitro phosphatase assay was adapted from [23]. Assays with and without inhibitor were performed in Corning 96-well clear bottom plates having a non-binding surface, with a total assay volume of 200 μL. The assay buffer contained 100 mM Tris, 100 mM NaCl, 1 mM EDTA at pH 7.0 and 2.2 mM *p*NPP as the substrate. Serial dilutions of each of the RR601 and NSC-87877 stocks were performed such that upon the addition of 4 μL of a respective inhibitor stock to the assay buffer, the resulting range in assay inhibitor concentrations were 0.03 μM to 100 μM for RR601 and 0.1 μM to 300 μM for NSC-87877. The reaction was initiated upon addition of 4 μL of a 5.0 μM PTP1B stock, which was dispensed to each of the wells except the blanks using a single channel pipette. The resulting PTPB1 concentration in the assay buffer was 0.1 μM. The plate was returned to the plate reader, shaken, and absorbance recorded at 25 °C every thirty seconds over a 10 mins period when RR601(Fig. 8a) was used, or for 60 mins when NSC-87877 was used as the inhibitor (Additional file 1: Fig. S1A). Since NSC-87877 has also been reported to inhibit SHP-2, we also examined the capacity of RR601 and NSC-87877 to inhibit SHP-2 (Creative Biomart). Assays were performed in 96-well plates in a similar manner as reported above. The assay buffer contained 50 mM HEPES (pH 7.4), 2 mM EDTA, 3 mM DTT and 100 mM NaCl. An initial experiment was performed to determine the kinetic parameters of the SHP-2 enzyme. The reaction was initiated with the addition of 4 μL of a 0.35 μM stock SHP-2 enzyme into wells containing 0, 1, 3, 9, 27 and 81 μM *p*NPP. The SHP-2 assay concentration was 7 nM. Absorbance was recorded every 30 s over a ten minute assay period at a temperature of 25 °C (Additional file 1: Fig. S8). Initial velocities were fit to the Michaelis-Menten equation:

1 $$ v=\frac{V_{m ax}\left[ S\right]}{K_m+\left[ S\right]} $$

where *v* is the initial velocity, *V*_*max*_ the maximum velocity, *K*_*m*_ the Michaelis constant, and [*S*] the *p*NPP concentration. Following the determination of the Michaelis constant of the SHP-2 enzyme in the *p*NPP assay, inhibition studies with RR601 and NSC-87877 were performed over concentration ranges of 0.01–100 μM and 0.03–300 μM, respectively. Assay buffer contained 7.5 mM *p*NPP, roughly one-half the value of the Michaelis constant determined from the study above. Reactions were initiated with the addition of 4 μL SHP-2 enzyme, resulting in a final assay enzyme concentration of 7 nM. Absorbance was recorded every 30 s over a 10 min reaction period at 25 °C in the RR601 study and every 30 s over a 60 min reaction period with NSC-87877.

### IC_50_ calculation

Initial rate values obtained from the plate reader were normalized to percent activity relative to the mean negative control rates. Graphpad Prism 6 software was utilized to calculate a non-linear regression (curve fit) using a variable slope model equation, constraining the top and bottom values to 100% activity and 0% activity, respectively, using the following equation:

2 $$ {v}_i= Bottom+\frac{\left( Top- Bottom\right)}{1+{10}^{\left( logI{C}_{50}- x\right)* Hill\  Slope}} $$

where *v*_*i*_ is the initial rate.

### Nephelometry

Nephelometry was performed to determine the relative propensity of the inhibitor compounds to aggregate in solution, based on the light scattering properties of the molecular aggregates. Compound aggregation can lead to artifact inhibitory effects, thus confounding a study of mechanism of inhibition. Compounds were tested for aggregation in a 96-well plate format, final volume 200 μL, using the phosphatase activity assay buffer at pH 7.5 without added *p*NPP. Compound concentrations ranging from 1 μM to 300 μM were generated by the addition of 4 μL volumes of serially diluted compound samples that were prepared from stock solutions of RR601, or RR535 and MP Biomedical stock solutions that had either been stored in the dark or exposed to light for 17 days. Eight wells were used for blanks and for each compound concentration. Plates were analyzed at two separate gains to determine if the signals were at saturating levels. Progesterone was used as a positive control for compound aggregation. Data were collected using a BMG NEPHELOStar Plus, equipped with a 635 nm laser.

### Mechanism of DUSP5 PD(WT) inhibition by RR601

To investigate the mechanism of DUSP5 PD(WT) inhibition by RR601, initial velocity inhibition profiles of RR601 were obtained in a 96-well plate format by measuring DUSP5 PD(WT) initial velocities in assay buffer containing of 1, 3, 9, 27 and 81 mM *p*NPP and 0, 3, 10, 30 and 75 μM RR601 at each *p*NPP concentration. Reactions were initiated by the addition of DUSP5 PD(WT) to a final concentration of 0.6 μM in each of the wells, excluding the blank. The data were fitted to a global competitive inhibition model (Graphpad Prism) using the following equation:

3 $$ {v}_i=\frac{V_{m ax}\left[ S\right]}{K_m\left(1+\frac{\left[ I\right]}{K_i}\right)+\left[ S\right]} $$

where *v* is the initial velocity, *V*_*max*_ the maximum velocity, *K*_*m*_ the Michaelis constant, [*S*] the concentration of *p*NPP, [*I*] the concentration of RR601 and *K*_*i*_ the inhibition constant.

The mechanism of RR601 inhibition of SHP-2 was investigated in a similar manner. Initial velocities of SHP-2 were determined in assay buffer containing 1, 2, 3, 10 and 30 mM *p*NPP along with 0.1, 0.3, 1, 2 and 3 μM RR601 at each *p*NPP concentration. The data were fitted to a global competitive inhibition model (Additional file 1: Fig. S9). For substrate concentrations less than 30 mM, the data fit best to a competitive inhibition model (Eq. 3).

### Spectroscopic study of the effect of light exposure on compound properties

It was observed that solutions of 1-amino-5-naphthol-7-sulfonic acid (originally NCI2602) prepared from solid material procured from different sources were different in color. Therefore, compound solutions were studied spectrophotometrically. In addition, it was also observed that solutions of RR535 and MP Biomedicals stored on the bench top became darker with time. This prompted a more systematic examination of the time-dependent nature of the color change. Freshly prepared 15 mM solutions of RR535 and MP Biomedicals were prepared in DMSO, from which 0.86 mM solutions in DMSO were prepared. Samples of each 0.86 mM solution were added to quartz cuvettes and the absorbance of each sample was recorded from 400 nm to 700 nm (Hewlett Packard 8456 diode array spectrophotometer). Each 0.86 mM sample was then divided into two equal volumes, one volume of each was stored in a cryovial on the benchtop exposed to room light while a second volume of each was stored in a cryovial wrapped in foil and placed in a drawer protected from light. Absorbance was recorded seven days later, after which the samples were returned to their respective bench top or drawer locations, and again at seventeen days following sample preparation. The remaining 15 mM RR535 and MP Biomedicals samples were stored on the bench top for seventeen days after which their inhibitory capacity against DUSP5 PD(WT) activity was determined.

### Additional analytical measurements

^1^H NMR spectra of the MP Biomedicals, RR535 and RR601 compounds were collected for comparison. Also, mass spectrometry (MALDI) of methanol extracts of MP Biomedicals and RR535 compounds were performed using a nitroanthracene matrix.

## Results

### Initial identification of NCI2602

**Fig. 1:**
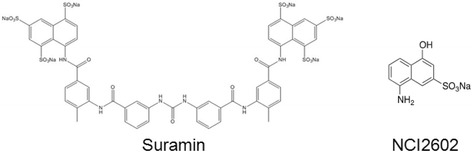
Chemical structures of suramin and NCI2602

**Fig. 2:**
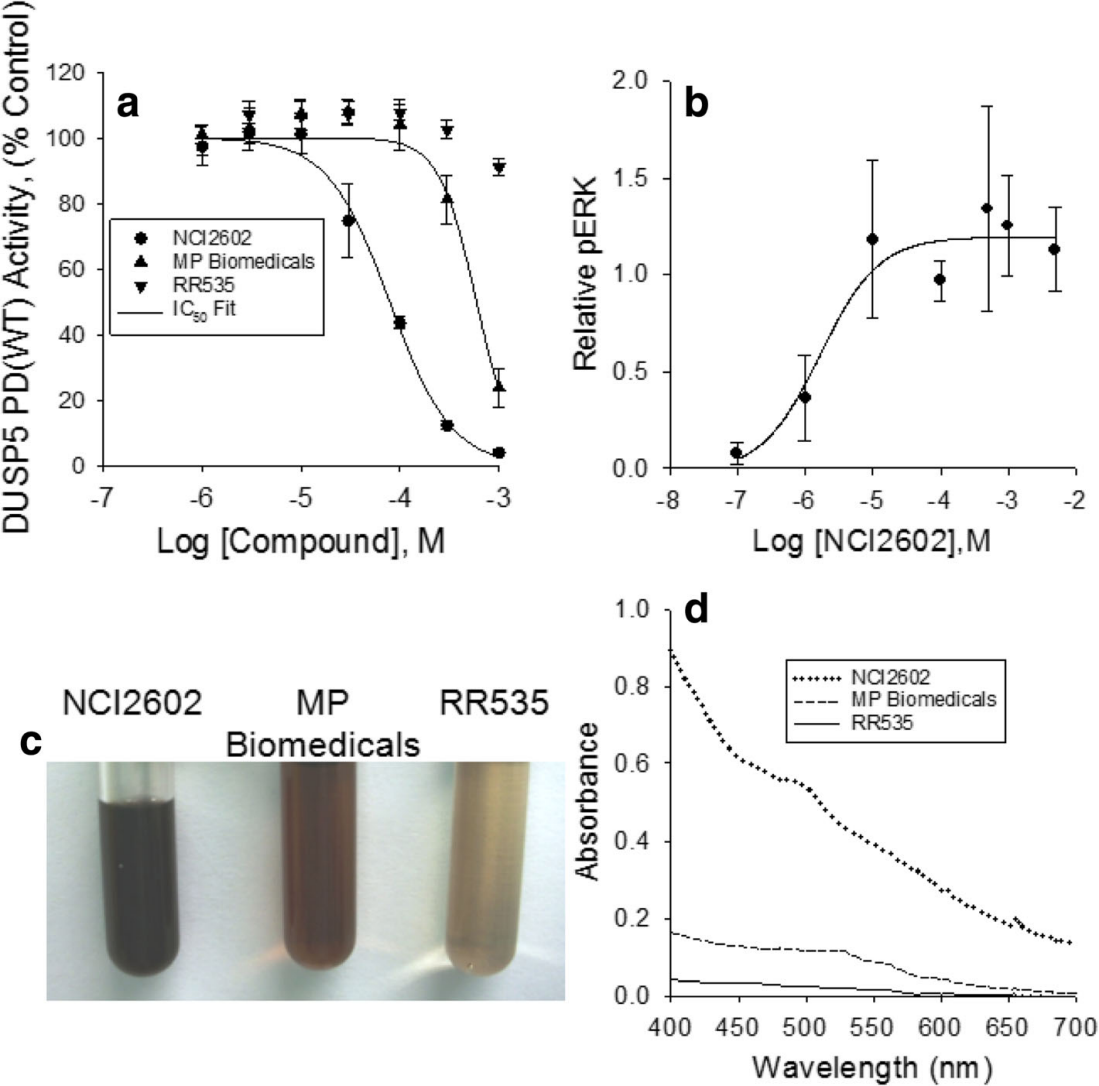
NCI2602 IC_50_ determinations for DUSP5 PD(WT) and DUSP5(WT) activities using *p*NPP and *p*ERK as substrates, respectively. **a** DUSP5 PD(WT) initial velocity versus increasing concentrations (1 to 1,000 μM) of NCI2602, MP Biomedicals and RR535 (two additional sources of NCI2602), using *p*NPP as the substrate. Lines represent the data fit to Eq. 1 resulting in calculated IC_50_ values of 78.5 ± 5.4 μM and 593.5 ± 64.1 μM (calculated IC_50_ value ± SE) for NCI2602 and MP Biomedicals, respectively. The model was unable to fit the RR535 data (did not converge) at the assayed concentrations. Data points represent the mean ± SD of three or four trials, with four to eight wells at each compound concentration. **b** Relative DUSP5(WT) activity versus increasing concentrations of NCI2602 utilizing *p*ERK as the substrate. The data points, generated from normalized image intensities, represent the mean ± SD of three experiments. A global model fit of the three data sets resulted in an estimated IC_50_ (± SE) value of 1.7 ± 1.2 μM. **c** Photographic images of 25 mM stock concentrations of NCI2602, MP Biomedicals and RR535 used for the IC_50_ determinations in (**a**). **d** Absorbance spectra from 400 to 700 nm of 1.8 mM concentrations of NCI2602, MP Biomedicals and RR535 in DMSO with DMSO as the blank

In our previous work, we had identified Suramin (Fig. 1), an FDA-approved compound that inhibited the DUSP5 enzyme [22]. Suramin was previously reported as an active site-directed, reversible, and tight binding inhibitor of protein-tyrosine phosphatases [24], and is an FDA approved drug for treatment of African trypanosomiasis [25]. Because Suramin has off target effects [26], and aggregates [22], we investigated the individual chemical moieties that comprise Suramin, and first procured a compound from the NCI Diversity Chemical Library (NCI2602) that resembled this moiety (Fig. 1). We first investigated the ability of NCI2602 to inhibit DUSP5 in our published *p*NPP and *p*ERK assays [21]. In the *p*NPP assay (Fig. 2a), we observed an IC_50_ of 78.5 ± 5.4 μM (± SE), while in our *p*ERK western blot assay (Fig. 2b), we observed an IC_50_ value of 1.7 μM ± 1.2 μM, as the average obtained from the global fit of three separate experiments. These data suggest that NCI2602 is a moderately potent inhibitor of DUSP5 activity. To confirm our findings above, we resynthesized NCI2602, referred to as RR535, and also purchased compound from a commercial vendor (MP Biomedicals, 05211488, CAS 489-78-1) and re-analyzed these compounds which should, in theory, be identical to NCI2602 and behave the same in the DUSP5 PD(WT)-*p*NPP and DUSP5-*p*ERK assays. Surprisingly, the compound obtained from MP Biomedicals and the resynthesized NCI2602 (RR535) were found to be less effective inhibitors than the originally discovered NCI compound (Fig. 2a). The MP Biomedicals compound IC_50_ was 593.5 ± 64.1 μM, while the RR535 compound failed to show inhibition over the concentration range tested. These data collectively suggest that NCI2602 and the purchased compound are not identical in structure, form and purity.

### Changes in NCI2602 color over time

To investigate the differences between NCI2602 and the resynthesized or purchased compound, we first focused on the color of these compounds from different sources. The NCI2602 was dark grey to black in color, the MP Biomedicals was modestly lighter and the synthesized RR535 was white in color. Photographic images of 25 mM samples of each compound in DMSO (Fig. 2c), as well as absorbance spectra of each compound (Fig. 2d) correlate with the observed color differences observed in the solids. We rationalized that the change in color could be associated with impurities in the NCI2602 preparations or light-induced changes that result in alterations in color. To address the later possibility, we incubated stock solutions of RR535 and MP Biomedicals in the dark or on the bench top exposed to room light.

**Fig. 3:**
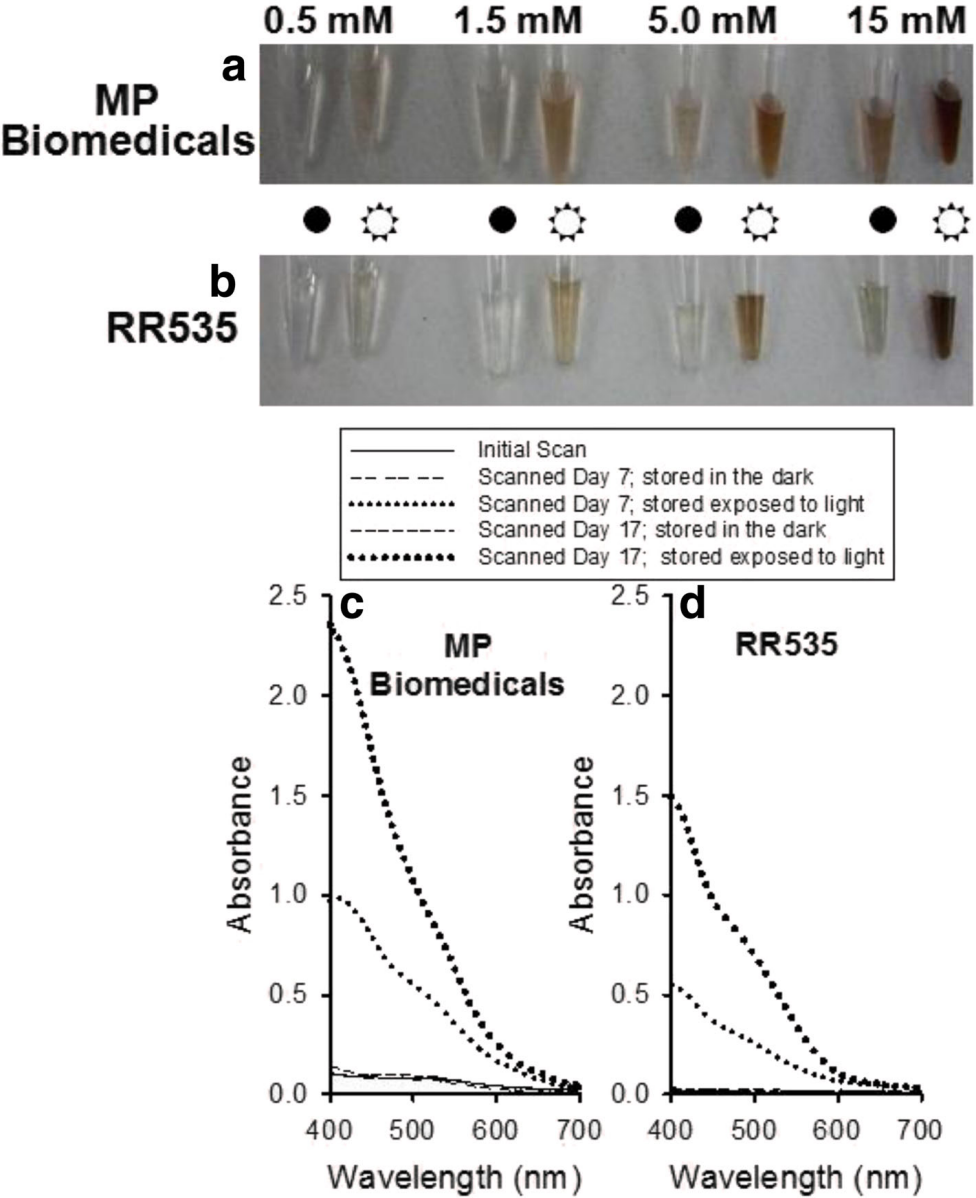
Light-sensitive nature of MP Biomedicals and RR535 compounds (1-amino-5-naphthol-7-sulfonic acid). Photographic images of two sets of (**a**) MP Biomedicals and (**b**) RR535 compounds at concentrations of 0.5, 1.5, 5.0 and 15.0 mM in DMSO which were either stored in the *dark* (•) or on the bench top exposed to a twelve hour cycle of room *light *(☼) over a one week period. Light exposure resulted in darkening of both compounds relative to the matched sample sets which were stored in the dark. **c** Absorbance spectra from 400 to 700 nm of 0.86 mM concentrations of MP Biomedicals and **d**) RR535 that were either stored in the dark or exposed to room light for seven and seventeen days. Scans of each compound on the day they were prepared from solid compound are included for comparison, demonstrating the light- and time-dependent increase in absorbance of both compounds

Stock solutions (15 mM) of RR535 and MP Biomedicals were prepared in DMSO and serially diluted to 5.0 mM, 1.5 mM and 0.5 mM using DMSO. Half of each sample was pipetted into matched 0.6 mL polypropylene microcentrifuge tubes, generating two sets of serially diluted RR535 and MP Biomedicals solutions. One set of each was stored on the bench top while the matched set was stored in a drawer protected from light. Photographic images of the samples of the MP Biomedical samples (Fig. 3a) or the RR535 samples (Fig. 3b) following storage for 7 days on either the bench top or the drawer were collected. The samples showed evidence of light-induced darkening when compared to the paired samples that were stored protected from light. Absorbance spectra collected from 1.5 mM samples of MP Biomedicals (Fig. 3c) and RR535 (Fig. 3d) stored for 7 and 17 days, diluted with DMSO to a final concentration of 0.86 mM, displayed a light- and time-dependent increase in absorbance over the visible range. These results suggest that color of the resynthesized and the purchased compound changed over time to light exposure, and matched the NCI2602 compound color.

### Effect of light exposure on RR535 and MP Biomedicals on *in vitro* potency: DUSP5 PD(WT) activity assays

**Fig. 4:**
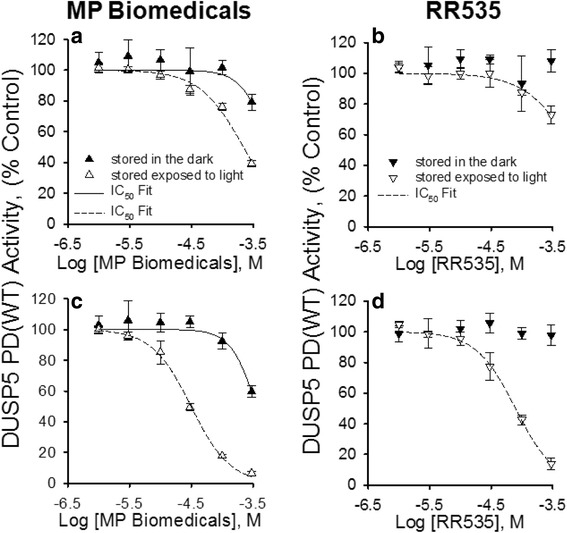
Effect of light exposure and assay reaction time on MP Biomedicals and RR535 IC_50_ values for DUSP5. DUSP5 PD(WT) initial velocity, monitored utilizing *p*NPP as the substrate, versus increasing concentrations of (**a**) MP Biomedicals and B) RR535 (1 to 300 μM) prepared from stock solutions that were either stored in the dark or exposed to room light for 17 days. IC_50_ values were determined from rate data collected during the first ten minutes of the reaction period. The calculated IC_50_ ± SE for MP Biomedicals that was stored in the dark was 588 ± 351 μM, compared to 221 ± 11 μM for the same compound when stored exposed to room light (**a**). RR535 that was stored in the dark did not inhibit DUSP5 PD(WT) activity over the concentration range tested, while the IC_50_ for light-exposed RR535 was 725 ± 206 μM (**b**). **c** MP Biomedicals IC_50_ values determined as in (**a**) from rate data collected between 15 and 25 min during the reaction period for compound stored in the dark or exposed to room light were 360 ± 36 μM and 31 ± 1 μM, respectively. **d** RR535 IC_50_ values determined as in (**b**) from rate data collected between 15 and 25 min. Compound stored in the dark did not inhibit DUSP5 PD(WT) activity while light exposed RR535 had an IC_50_ of 78.8 ± 4.9 μM. Data points represent the mean ± SD of four trials with four wells at each MP Biomedicals and RR535 concentration

The observed color changes brought about by light exposure led us to examine whether light exposure impacted the inhibitory capacity of MP Biomedicals and RR535 compounds with respect to DUSP5 PD(WT) activity. Figure 4 shows IC_50_ curves generated from DUSP5 PD(WT) activity versus increasing concentrations of MP Biomedicals (Fig. 4a) and RR535 (Fig. 4b) (1 to 300 μM) prepared from stock solutions that had either been stored in the dark or exposed to room light for 17 days, using *p*NPP as the DUSP5 PD(WT) substrate. The data shown in Fig. 4a and b were collected during the first 10 mins of the reaction period. The calculated IC_50_ values for the MP Biomedicals compound that had been stored in the dark or exposed to room light were 588 ± 351 μM and 221 ± 11 μM, respectively. RR535 that was stored in the dark did not inhibit DUSP5 PD(WT) activity over the concentration range studied, while RR535 compound exposed to room light had an IC_50_ value of 725 ± 206 μM. When DUSP5 PD(WT) activity was assayed between 15 and 25 mins during the reaction, the calculated IC_50_ values for the MP Biomedicals compound that had been stored in the dark or exposed to room light were 360 ± 36 μM and 31 ± 1 μM, respectively (Fig. 4c). RR535 that was stored in the dark did not inhibit activity over the concentration range tested, while the IC_50_ for light-exposed RR535 was 78.8 ± 4.9 μM (Fig. 4d) when assayed between 15 and 25 mins. There was no significant difference between the initial assay reaction rates of the negative controls recorded from 0 to 10 min or 15 to 25 min. These data collectively suggest that light induced changes in the activity associated with these compounds.

The exact nature of the light-induced change on the structure of RR535 was not known, but was hypothesized to involve the dimerization reaction shown below based on the fact that arylamines are known to undergo ready oxidation when exposed to air and light forming highly colored azo dyes. Accordingly, a deliberate light-induced transformation of pure RR535 was carried out in the presence of air (see Methods section) and the structure of the resulting dimeric azo dye (RR601) was established by mass spectrometry and NMR spectroscopy.

This newly synthesized compound was called “RR601” and was tested as an inhibitor of DUSP5.

**Fig. 5:**
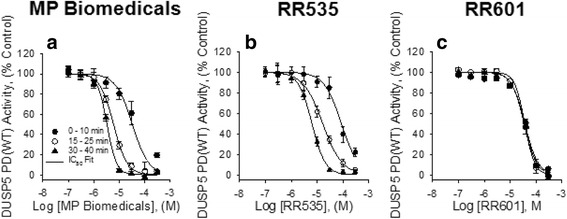
Light-exposed MP Biomedicals and RR535 inhibit DUSP5 PD(WT) in a time-dependent manner while inhibition by RR601 is not time-dependent. DUSP5 PD(WT) initial velocity monitored utilizing *p*NPP as the substrate versus increasing concentrations of **(a**) MP Biomedicals, (**b**) RR535 (0.1 to 300 μM) prepared from compounds exposed to room light for 90 days, and (**c**) freshly prepared RR601 (0.1 to 300 μM). IC_50_ ± SE values were determined from rate data collected during the first ten minutes, between 15 and 25 min and again between 30 and 40 min during the reaction period (Table 1). Data points represent the mean ± SD of three trials with eight wells at each compound concentration

Because we observed increased activity over time for the original monomeric inhibitors (NCI2602, RR535 and MP Biomedicals), we performed a time-dependent light incubation profile for these compounds. Inhibition as a function of time exposed to light in the plate reader (Fig. 5) shows changes for DUSP5 PD(WT) inhibition activity for MP Biomedicals (Fig. 5a), RR535 (Fig. 5b), and RR601 (Fig. 5c) inhibitors. MP Biomedicals and RR535 compounds were prepared from stock solutions exposed to light for 90 days while the RR601 stock was prepared from recently synthesized compound. Compounds were tested for their ability to inhibit DUSP5 PD(WT) activity in the plate assay and IC_50_ values calculated from dose response curves generated from activity data collected over the first 10 mins of the assay reaction period, then from 15 to 25 mins and from 30 to 40 mins, respectively (Table 1). IC_50_ values for MP Biomedicals and RR535 decreased with time however, RR601 was unique in that its ability to inhibit did not change as a function of time exposed to the light source in the plate reader.

**Table 1:** DUSP5 PD(WT) inhibitor IC_50_ values with time

	*Assay Reaction Period*		
*Inhibitor Compounds*	0 – 10 min	15 – 25 min	30 – 40 min
MP Biomedicals	32.5 ± 2.8	5.9 ± 0.2	3.3 ± 0.2
RR535	86.6 ± 8.0	16.5 ± 0.7	6.3 ± 0.2
RR601	36.9 ± 3.9	36.5 ± 1.5	34.5 ± 1.4

IC_50_ values (± SE) for MP Biomedicals, RR535, and RR601 were determined in the presence of DUSP5 PD(WT) as described in Fig. 4. Data was collected during three successive 10 min assays with *p*NPP as the substrate

We also performed *p*ERK assay with RR535 and RR601 compounds (Additional file 1: Fig. S3 and Additional file 1: Fig. S4), and found that indeed they inhibit DUSP5 activity with IC_50_’s 22.8 μM for RR535 and 4.8 μM for RR601. Thus, collectively our results suggest that the azo-bridged dimer of RR535, referred to as RR601, is actually the more potent inhibitor of DUSP5.

### Nephelometry measurements of RR535, MP Biomedicals and RR601 aggregation

**Fig. 6:**
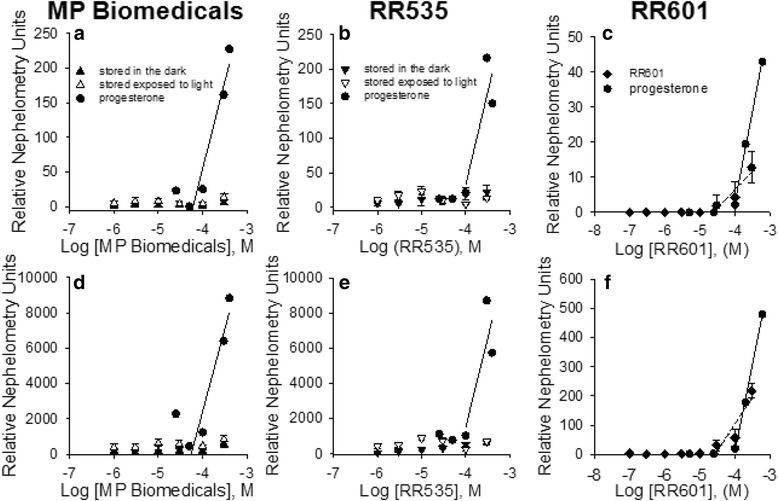
MP Biomedicals, RR535, and RR601 Nephelometry at two gain settings. Aggregation of MP Biomedicals (**a** and **d**), RR503 (B and E) and RR601 (C and F) was measured in 96 well plates with increasing concentrations of compound at gain levels of 35 (**a**, **b**, and **c**), and 50 (**d**, **e**, and **f**). MP Biomedicals and RR535 samples were prepared from stock solutions that were either stored in the dark or exposed to room light for 17 days. Stock solutions of RR601 were stored in the dark. Progesterone (25 to 400 μM) was used as the control. Data points at each compound concentration are the mean ± SD of eight wells. Single wells were used for each progesterone concentration

Since compound aggregation is a common source of false positive inhibition, especially for polysulfonated aromatic compounds [27], nephelometry experiments were performed on all compounds (Fig. 6), to rule out this type of artifact inhibition. Nephelometry experiments were performed as described in our previous publication [22], to measure the propensity for RR535, MP Biomedicals and RR601 compounds to aggregate in solution. In all panels, the positive control compound progesterone is seen to aggregate at approximately 100 μM, and shown as an inflection in the curve. In Fig. 6 panels a, b, d and e no other inflection is observed; in contrast, several points begin to show light scattering in panels C and F. Figures 6b and e show nephelometry (compound aggregation) measurements for RR535 prepared from stock solutions that had been stored in the dark or exposed to room light for seventeen days. There was little or no measurable aggregation of either sample over the concentration range tested. Similarly, Fig. 6a and d show nephelometry measurements as a function of increasing concentrations of MP Biomedicals prepared from stock solutions that had been stored in the dark or exposed to room light for seventeen days. Here also, there was little or no measurable compound aggregation over the concentration range tested. In contrast, RR601 (Fig. 6c and f) began to aggregate at approximately 30 μM. Since aggregation occurs at a concentration that is higher than the IC_50_, inhibition is not due to aggregation effects.

### Mechanism of RR601-mediated DUSP5 PD(WT) inhibition

**Fig. 7:**
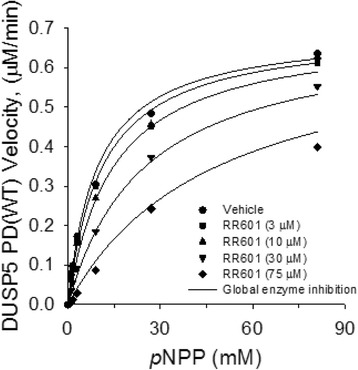
Global non-linear regression fit for competitive inhibition of DUSP5 PD(WT) with RR601. DUSP5 PD(WT) reaction velocities were measured in assay buffer containing 1, 3, 9, 27 and 81 mM *p*NPP in the presence of 0, 3, 10, 30 and 75 μM RR601. The data was fit with GraphPad curve fitting software. Best fit estimates (± SE) for V_max_, K_m_ and K_i_ are 0.70 ± 0.02 μM · min^-1^, 9.6 ± 0.9 mM and 18.8 ± 2.5 μM, respectively

RR601 inhibition of DUSP5 PD(WT) with *p*NPP as the substrate showed no time dependence (Fig. 5c). RR601 was shown to be a competitive inhibitor versus DUSP5 PD(WT), in initial velocity studies varying concentrations of *p*NPP and RR601. A global competitive inhibition fit was performed, fitting the data to Eq. 3 (Fig. 7), resulting in best fit estimated values (± SE) for *V*_*max*_ (0.70 ± 0.02 μM · min^-1^), *K*_*m*_ (9.6 ± 0.9 mM) and *K*_*i*_ (18.2 ± 2.5 μM). A Lineweaver-Burk double reciprocal plot of the data (Additional file 1: Fig. S5) was also consistent with a competitive inhibition mechanism.

### RR601- and NSC-87877-mediated PTP1B and SHP-2 inhibition

**Fig. 8:**
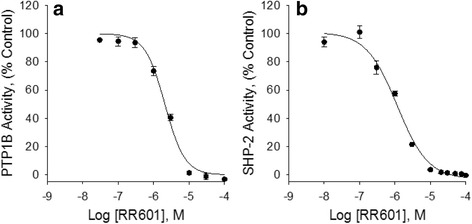
RR601 inhibits PTP1B and SHP-2. PTP1B and SHP-2 initial velocities versus RR601 concentration were fit to Eq. (1) resulting in estimated RR601 IC_50_ ± SE values of 2.1 ± 0.2 for PTP1B and 1.1 ± 0.1 μM for SHP-2. Activity was monitored utilizing *p*NPP as the substrate. Data points represent the mean ± SD of three or four trials with three to eight wells at each RR601 concentration

**Fig. 9:**
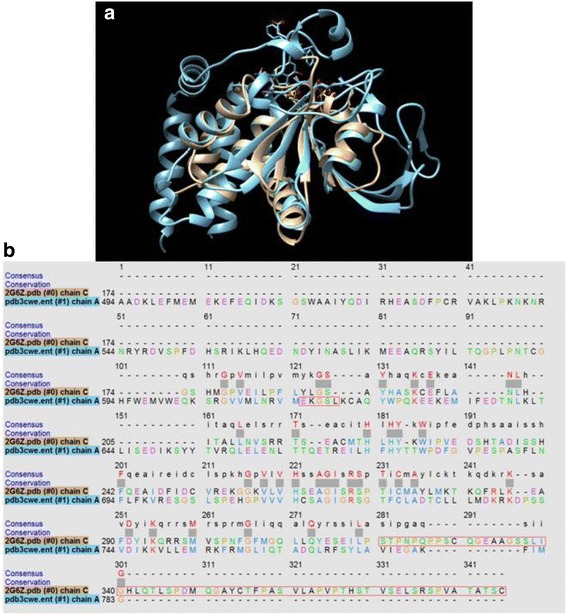
Comparison of PTP1B and DUSP5 PD. **a** Structural overlay of PTP1B and DUSP5 PD(WT), based on crystal structures with pdb codes 3CWE (PTP1B with a phosphonic acid inhibitor bound) and 2G6Z (DUSP5 PD), using Chimera. **b** Primary sequence alignment was done using Clustal Omega pairwise alignment and guide tree algorithm [37], which indicated an extremely low percent identity of 9.5%

On searching the literature and compound databases, we noticed chemical structure similarities between NSC-87877 (a reported inhibitor the tyrosine phosphatases SHP2 (IC_50_ = 0.3 μM) and PTP1B (IC_50_ = 1.7 μM) [28]) and RR601, which inhibits DUSP5 PD(WT) (IC_50_ = 36 μM). This observation prompted us to investigate whether the newly synthesized RR601 would also inhibit PTP1B and SHP-2. Figure 8 show PTP1B and SHP-2 activity versus increasing concentrations of RR601 using *p*NPP as the substrate, and indicate surprisingly strong inhibition of PTP1B and the related phosphatase, SHP-2 activity by RR601. The IC_50_ (± SE) values determined from the fitted curves are 2.1 ± 0.2 μM and 1.1 ± 0.1 μM in the presence of PTP1B and SHP-2, respectively. These values are significantly below the aggregation point of 30 μM for RR601, based on nephelometry. Thus, RR601 is a more potent and selective inhibitor of PTP1B (2 μM) and SHP-2 (1 μM) than it is for its originally intended target, DUSP5 (36 μM). PTP1B and DUSP5, while sharing the mechanism of being cysteine-based phosphatases, have very different tertiary and primary structures (Fig. 9). Thus, while the discovery that RR601 is a more potent inhibitor of PTP1B than of DUSP5 was serendipitous, it is not surprising that 18-fold selectivity could be obtained for PTP1B over DUSP5, given these structural differences.

Interestingly, NSC-87877 in the same assay did not initially inhibit PTP1B activity (Additional file 1: Fig. S1A) at the tested concentrations; however, continuous monitoring of the assay over time revealed IC_50_ values of 723 ± 302 and 337 ± 130 μM after 50 and 60 mins, respectively, of the reaction assay. Also, NSC-87877 did inhibit SHP-2 activity with an IC_50_ value of 168 ± 30 μM, with no change in the IC_50_ value after 60 mins (Additional file 1: Fig. S1B). NSC-87877 did not inhibit DUSP5 PD(WT) activity (Additional file 1: Fig. S1C). Collectively, these data suggest that RR601 shows more selectivity for PTP1B and SHP-2 over DUSP5, similar to the less potent NSC-87877.

## Discussion

Protein tyrosine phosphatases (PTPs) are drug targets for a wide range of diseases, ranging from vascular anomalies and cancer (DUSP5) to diabetes (PTP1B) [4–16]. Unfortunately, PTPs are also extremely challenging targets for developing drugs, as evidenced by the fact that there are currently no FDA-approved drugs that target PTPs [16]. Furthermore, drug screening for PTP targets often produces false positive or false negative results. Studies presented herein address a false positive and negative result, wherein that actual active molecule is a product of light-induced chemical reaction formed *in situ*, or upon extended storage of a compound by the compound provider.

Our initial studies focused on identifying inhibitors of DUSP5, as drug lead molecules to treat vascular anomalies. Compounds were screened *in silico* first, via docking studies, followed by enzyme inhibition studies. A lead compound – NCI2602 – was identified from the National Cancer Institute (NCI) database, and then obtained and experimentally tested and found to be an inhibitor of DUSP5. But, the compound was observed to have variable potency depending on its source (NCI; commercial; internally synthesized; see Fig. 2). Furthermore, upon careful study of mechanism of inhibition, the compound was found to have a potency that increased over time, and only after exposure to light and oxygen. Such exposure led to a color change for the compound (Fig. 3), which correlated with increased potency (Fig. 4). Re-synthesis of the compound (referred to as RR535) led to the surprising result that the in-house synthesized compound actually had little enzyme inhibition activity, compared to compounds from other sources (Fig. 2a). But, after exposure to light and oxygen, it acquired enzyme inhibition activity. Thus, we speculated that the compound underwent a chemical modification *in situ*, and that modification was identified as a light-induced dimerization to produce the azo-bridge sulfonated napthol, RR601. This hypothesis was tested by synthesizing and testing the dimeric RR601 compound, which was found to be much more potent than the monomeric compound (RR535 or NCI2602) that was initially screened. Since sulfonated compounds are known to produce false positive inhibition via aggregation effects [27], it was of course possible that RR601 was not a true inhibitor. But, based upon nephelometry experiments, the sulfonated compounds tested – including RR601 – showed less propensity for aggregation relative to the control aggregator progesterone (Fig. 6). Thus, the RR601 dimeric compound is a relatively potent inhibitor of DUSP5 activity, with an IC_50_ of 36 μM. Proton NMR (Additional file 1: Fig. S6) and mass spectrometry (Additional file 1: Fig. S7) revealed differences between the MP Biomedicals and RR535 compounds. Careful control experiments to monitor compound stability, coupled with re-synthesis of the original compound, and re-synthesis of the proposed *in situ* reaction product, was crucial for identifying the actual lead molecule (RR601), which is not the compound structure that was advertised by the suppliers. The above results provide a cautionary lesson on the importance of verifying compound identity using chemical re-synthesis.

RR601 had modest potency as a DUSP5 inhibitor, our originally intended drug target. But, its structural similarity to NSC-87877 implied that RR601 could target other cysteine phosphatases within the class I PTPs, such as PTP1B, a known target of NSC-87877. PTP1B is a widely pursued target for treating diabetes [15]. These two PTPs, while in the same family and having similar mechanisms, have very little structural similarity (Fig. 9), suggesting it should be possible for an inhibitor to inhibit one selectively over the other. Testing RR601 against PTP1B, for selectivity, demonstrated that the dimeric RR601 was actually a more potent inhibitor of PTP1B than of DUSP5, with an IC_50_ of 2.1 μM against PTP1B. Interestingly, RR601 is an azo-bridged dimer of sulfonated naphthol rings, and resembles previously reported PTP inhibitors [28, 29], but with 18-fold selectivity for PTP1B. Since SHP-2 is also a reported target of NSC-87877, we tested RR601 against SHP-2 and determined an IC_50_ of 1.1 μM. RR601 is therefore selective versus DUSP5, but shows no selectivity between the Class 1 phosphatases, PTP1B and SHP-2. Thus, we have discovered a new molecule, in an established class of PTP1B (Class 1 phosphatase) inhibitors, by serendipity. In particular, our path to a potent and selective PTP1B inhibitor began by first targeting DUSP5, and screening a commercial library. Through a series of control experiments, we discovered one weak inhibitor that displayed light- and time-dependent inhibition of DUSP5, due to *in situ* formation of a colored and more active form of inhibitor. Based on a chemical understanding of the potential light-induced reactivity of the compound in question, it was demonstrated that the compound formed an azo-bridged dimer. Such dye-like compounds, comprised of polysulfonated aromatic rings tethered by an azo bridge, are actually well-known PTP inhibitors [28, 29]. In fact, a recent study of food dyes in this class revealed that half of such compounds were actually PTP inhibitors, with several inhibiting in the low micromolar range [29]. Of particular interest, compound NSC-87877 (see Additional file 1: Table S1) bears some resemblance to the azo-bridged polysulfonated aromatic PTP inhibitor discovered herein, and has been reported as a potent and selective inhibitor (300 nM) of the SHP-2 PTP [28]. Although, it should be noted that our data indicate that NSC-87877 may be less potent than previously reported, and is less potent that the RR601 compound reported herein.

Importantly, the active compound we identified in our screening efforts was not the structure advertised by the supplier; however, it is still a unique and valuable lead molecule. Our results therefore illustrate the importance of verifying compound identity in drug discovery efforts, and add another cautionary note in the growing concerns being expressed over reproducibility of published research studies, particular for preclinical drug development work [30, 31]. A study by Amgen scientists of published academic research in oncology found that <50% of published studies could be reproduced [31], with similar results obtained in a report compiled by researchers at Bayer [31, 32]. Some of these inconsistencies could be attributed to false positives that can occur due to chemical contaminants that are nonspecific and reactive [33, 34]. In others, results like what we report here could explain the discrepancies. Mis-assignment of chemical structure (i.e. error in identifying what a chemical structure actually is) can also occur for compounds that show up as false positive or negative hits in screening campaigns, because inhibitors are only formed *in situ* during the screening process – as we have observed in the studies reported herein. Such issues can have serious consequences. For example, an anti-cancer compound was patented and approved for human clinical studies; but, the Janda lab at Scripps later showed the chemical structure of that drug lead molecule had actually been mis-assigned [35]. The wrong molecule had been patented and approved for clinical studies. Although this is an extreme case, the problem of compound identity mis-assignment in drug discovery and development is considered to be common when high throughput screening methods are used [36]. Our take home lesson from our screening efforts is that compound structure verification by NMR or other methods, followed by characterization of physical properties, re-synthesis and re-testing against the original target is a must. The value of comparative structure assessments is underappreciated, and perhaps can pivot drug discovery efforts in a new direction, as was the case here. Collectively, our studies here point to a flexible, and highly dynamic model for drug discovery that encompasses multiple labs, orthogonal complementary approaches, and keen observation with deductive reasoning as key elements to making drug discovery valuable and fun in academics.

## Conclusion

Studies presented herein provide lessons on the importance of verifying mechanism of inhibition and compound identity when performing screening campaigns to identify enzyme inhibitors; and, on the value of being open to serendipity. Compound re-synthesis and verification was used to diagnose and characterize the *in situ* dimerization of a DUSP5 enzyme inhibitor, resulting in the serendipitous discovery of a new lead molecule for inhibiting both DUSP5 and PTP1B. Initial docking and then enzymatic screening of compounds from the National Cancer Institute (NCI) resulted in identification of an inhibitor of DUSP5 that showed time-dependent inhibition, with an IC_50_ of 36 μM. The active form of the compound was shown to be an azo-bridged dimer of sulfonated naphthol rings, which formed upon exposure to light. It behaves as a competitive inhibitor of DUSP5; and, is an even more potent inhibitor of PTP1B, with an IC_50_ of 2.1 μM.

## Additional file

Additional file 1: Figure S1. NSC-87877 IC_50_ determinations for PTP1B, SHP-2 and DUSP5 PD(WT). **Figure S2.** Inhibition of GST-DUSP5-mediated *p*ERK dephosphorylation by NCI2602. **Figure S3** ***.*** Inhibition of GST-DUSP5-mediated *p*ERK dephosphorylation by RR535. **Figure S4.** Inhibition of GST-DUSP5-mediated *p*ERK dephosphorylation by RR601; experiment as in Fig. S3. **Figure S5.** Lineweaver-Burk double reciprocal plot of inverse DUSP5 PD(WT) velocity versus inverse *p*NPP concentration in the presence of various RR601 concentrations. **Figure S6.** Proton NMR spectra of MP Biomedicals, RR535 and RR601. **Figure S7.** Mass spectrometry of MP Biomedicals and RR535 compounds. **Figure S8** ***.*** Michaelis-Menten kinetics for SHP-2 enzyme with *p*NPP as the substrate. **Figure S9.** Global non-linear regression fit for competitive inhibition of SHP-2 with RR601. (DOCX 321 kb)

## Abbreviations

DMSO: Dimethyl sulfoxide
DTT: Dithiothreitol
DUSP5: Dual specificity phosphatase 5
EDTA: Ethylenediaminetetraacetic acid
ERK2: Extracellular-regulated kinase 2
GST: Glutathione-S-transferase
MAPK: Mitogen-activated protein kinase
NMR: Nuclear magnetic resonance
PD: Phosphatase domain
*p*ERK: Phosphorylated extracellular-regulated kinase
*p*NPP: *p*-Nitrophenylphosphate
PTP: Protein tyrosine phosphatase
SHP-2: Protein tyrosine phosphatase, non-receptor type 11
WT: Wild type
