# Supplementary material for: Serendipitous discovery of light-induced (In Situ) formation of an Azo-bridged dimeric sulfonated naphthol as a potent PTP1B inhibitor
Source: BMC Biochem. 2017 May 31;18:10. doi: 10.1186/s12858-017-0083-3 (PMC5452347; doi:10.1186/s12858-017-0083-3)
Supplement: Additional file 1: Figure S1. — NSC-87877 IC50 determinations for PTP1B, SHP-2 and DUSP5 PD(WT). Figure S2. Inhibition of GST-DUSP5-mediated pERK dephosphorylation by NCI2602. Figure S3 . Inhibition of GST-DUSP5-mediated pERK dephosphorylation by RR535. Figure S4. Inhibition of GST-DUSP5-mediated pERK dephosphorylation by RR601; experiment as in Fig. S3. Figure S5. Lineweaver-Burk double reciprocal plot of inverse DUSP5 PD(WT) velocity versus inverse pNPP concentration in the presence of various RR601 concentrations. Figure S6. Proton NMR spectra of MP Biomedicals, RR535 and RR601. Figure S7. Mass spectrometry of MP Biomedicals and RR535 compounds. Figure S8 . Michaelis-Menten kinetics for SHP-2 enzyme with pNPP as the substrate. Figure S9. Global non-linear regression fit for competitive inhibition of SHP-2 with RR601. (DOCX 321 kb) [file 12858_2017_83_MOESM1_ESM.docx]

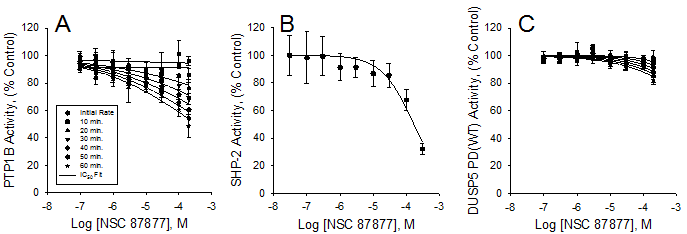


**Fig. S1.** *NSC-87877 IC_50_ determinations for PTP1B, SHP-2 and DUSP5 PD(WT).* A) PTP1B, B) SHP-2 and C) DUSP5 PD(WT) initial velocity versus increasing concentrations of NSC-87877 monitored over a period of 60 minutes. The estimated IC_50_ ± SE values for NSC-87877 at 60 minutes when mixed with PTP1B, SHP-2 and DUSP5 PD(WT) were 337 ± 130 µM, 168 ± 30 µM and > 3,000 µM, respectively.


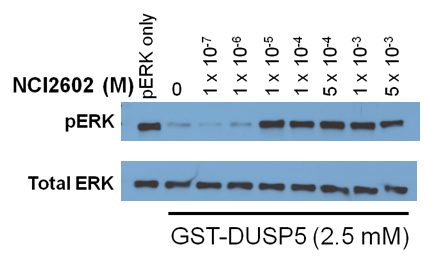


**Fig. S2.** *Inhibition of GST-DUSP5-mediated pERK dephosphorylation by NCI2602.* Western blot image generated following the reaction of active ERK2 with GST-DUSP5 samples that were incubated with 1 x 10^-7^ to 5 x 10^-3^ M NCI2602. In addition, active ERK samples without added GST-DUSP5 and samples with ERK and GST-DUSP5 but without NCI2602 were generated as controls. Total ERK is shown for comparison.


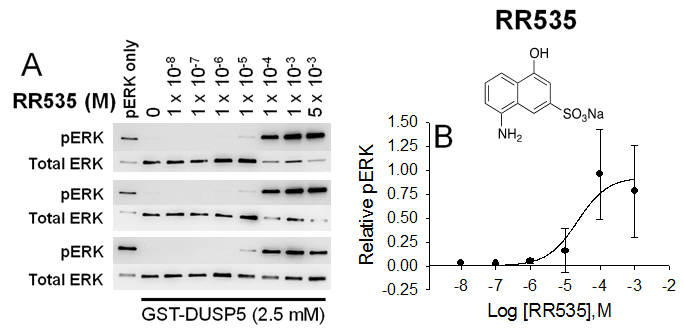


**Fig. S3.** *Inhibition of GST-DUSP5-mediated pERK dephosphorylation by RR535.* Panel A shows three Western blot images generated following the reaction of active ERK2 with GST-DUSP5 samples that were incubated with 1 x 10^-8^ to 5 x 10^-3^ M RR535. In addition, active ERK samples without added GST-DUSP5 and samples with ERK and GST-DUSP5 but without RR535 were generated as controls. Total ERK is shown for comparison. Pooled densitometry analysis of three separate experiments are shown in panel B.


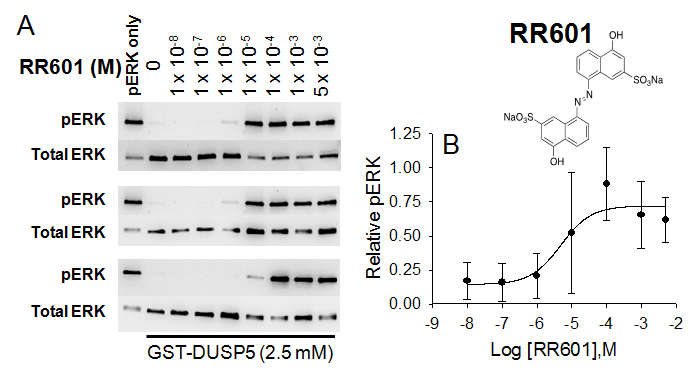


**Fig. S4.** *Inhibition of GST-DUSP5-mediated pERK dephosphorylation by RR601; experiment as in Additional file: Fig. S3.*


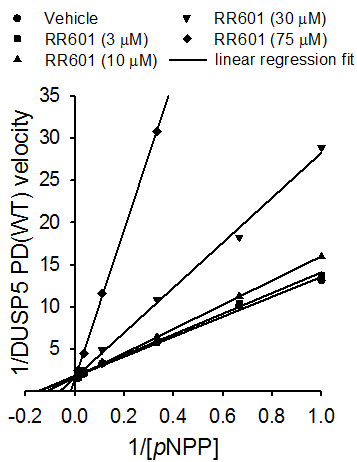


**Fig. S5.** *Lineweaver-Burk double reciprocal plot of inverse DUSP5 PD(WT) velocity versus inverse pNPP concentration in the presence of various RR601 concentrations.* *V_max_* values for vehicle, 3, 10, 30 and 75 μM RR601 are 0.57, 0.54, 0.61, 0.62 and 0.69 μM · min^-1^, respectively. *K_m_* values for vehicle, 3, 10, 30 and 75 μM RR601 are 6.8, 6.8, 8.7, 16.1 and 61.4 mM, respectively.


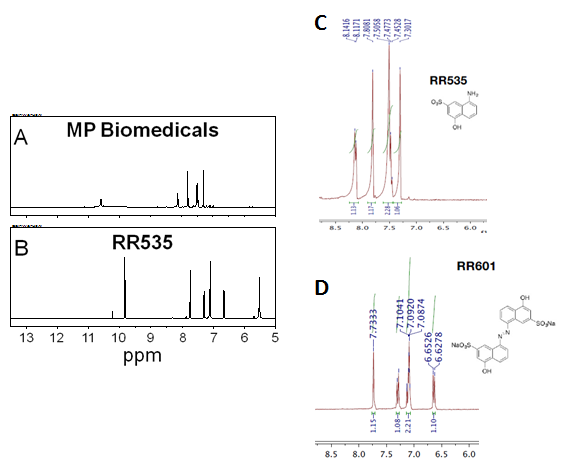


**Fig. S6**.  *Proton NMR spectra of MP Biomedicals, RR535 and RR601.*

**RR535:**

^1^H NMR (300 MHz, DMSO-*d*_6_) δ_H_ 10.61 (s, 1H), 8.13 (d, *J* = 7.4 Hz, 1H), 7.81 (s, 1H), 7.64-7.42 (m, 2H), 7.30 (s, 1H)

**RR601:**

^1^H NMR (300 MHz, DMSO-*d*_6_) δ_H_ 9.82 (s, 1H), 7.73 (s, 1H), 7.29 (d, *J* = 8.2 Hz, 1H), 7.17-7.07 (m, 2H), 6.64 (d, *J* = 7.4 Hz, 1H), 5.50 (s, 2H)

^1^H NMR (400 MHz, D_2_O) δ_H_ 7.92 (s, 1H), 7.67 (d, *J* = 8.3 Hz, 1H), 7.43 (t, *J* = 7.9 Hz, 1H), 7.18 (s, 1H), 7.02 (d, *J* = 7.5 Hz, 1H)


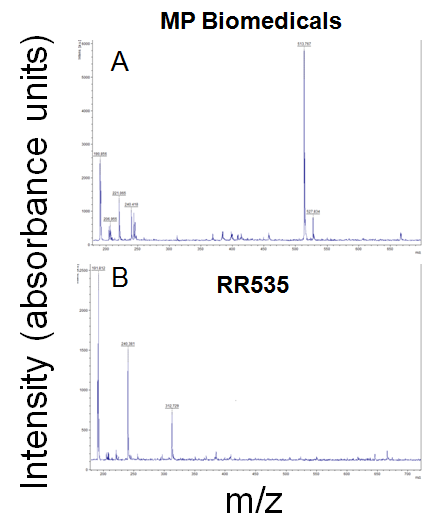


**Fig. S7.** *Mass spectrometry of MP Biomedicals and RR535 compounds.*


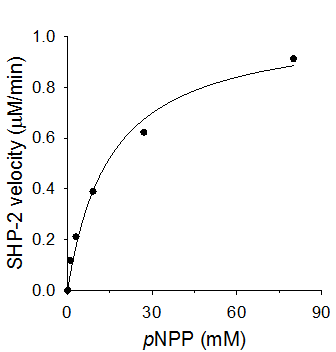


**Fig. S8.** *Michaelis-Menten kinetics for SHP-2 enzyme with pNPP as the substrate.* SHP-2 initial velocity versus pNPP concentration data was fit using equation 1. Model fit values for *V_max_* and *K_m_* (± SE) were 1.1 ± 0.1 μM · min^-1^ and 15.2 ± 3.1 mM, respectively.


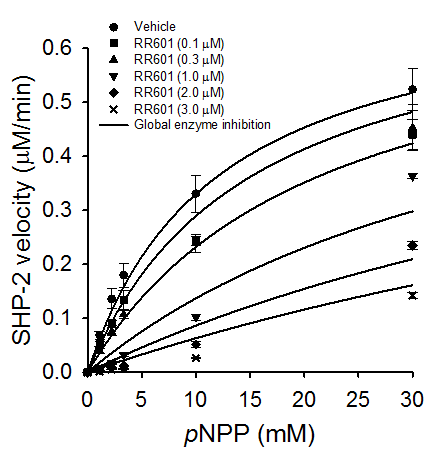


**Fig. S9.**  *Global non-linear regression fit for competitive inhibition of SHP-2 with RR601.* SHP-2 initial reaction velocities were measured in assay buffer containing 1, 2, 3, 10 and 30 mM pNPP in the presence of 0, 0.1, 0.3, 1.0, 2.0 and 3.0 μM RR601. Best fit estimates (± SE) for *V_max_*, 0.72 ± 0.04 μM · min^-1^; *K_m_*, 11.9 ± 1.7 mM and *K_i_* _,_ 0.38 ± 0.05 μM were obtained from a global competitive model fit (eq. 3). Data points represent the mean ± SD of three experiments.

| ***Compound*** | ***Structure*** |
| --- | --- |
| NCI2602  MP Biomedicals  RR535 | 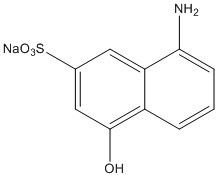 |
| RR601 | 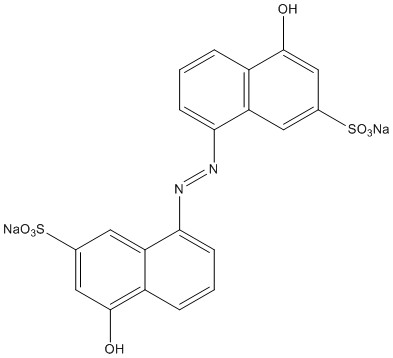 |
| NSC 87877 | 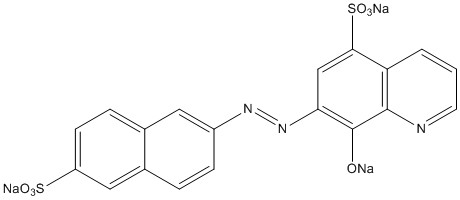 |

**Table S1.** *Inhibitor compound structures.*
